# Femoral migration of the cementless Oxford which caused the bearing dislocation: a report of two cases

**DOI:** 10.1186/s12891-020-03385-0

**Published:** 2020-06-08

**Authors:** Hiroshi Inui, Shuji Taketomi, Ryota Yamagami, Kohei Kawaguchi, Sakae Tanaka

**Affiliations:** Department of Orthopaedic Surgery, Faculty of Medicine, The University of Tokyo, 7-3-1 Hongo, Bunkyo-ku, Tokyo, 113-0033 Japan

**Keywords:** Unicompartmental knee arthroplasty, Oxford mobile bearing, Bearing dislocation, Cementless prosthesis, femoral migration

## Abstract

**Background:**

There are no previous reports on the complications around the femoral component of cementless Oxford unicompartmental knee arthroplasty (UKA). However, we experienced two cases of femoral migration to the proximal side, which caused bearing dislocations after cementless Oxford UKA.

**Case presentation:**

Case1. In an 82-year-old woman, bearing dislocation occurred 13 months postoperatively because of femoral migration that was resolved with an revision surgery to cemented component and thicker mobile insert.

Case2. In a 52-year-old man, first bearing dislocation occurred 7 months postoperatively. Five months after revising the insert to a thicker one, another dislocation occurred mainly because of the femoral migration. Eventually, a revision to total knee arthroplasty was necessary.

**Conclusions:**

The inferred main reasons of femoral migration of cementless Oxford were osteoporosis for the first case and early return to high performance sports activity for the second case. Although several merits of using cementless prosthesis, particularly better fixation and lesser radiolucency than cemented prosthesis, have been reported, surgeons should pay attention to the patient’s bone quality and advise a slow return to high-level physical activity.

## Background

Unicompartmental knee arthroplasty (UKA) is performed for the treatment of isolated unicompartmental knee disease. The Oxford Knee (Zimmer-Biomet, Swindon, United Kingdom), which features a fully congruent mobile bearing designed to minimize wear, has been in clinical use for > 40 years [1, 2]. Satisfactory results have been achieved with UKA in the medial compartment when strict inclusion criteria are followed [3, 4]. However, UKA is associated with a significantly higher rate of revision as reported by several studies [5, 6]. One of the reasons reported is the misinterpretation of radiolucent lines that are commonly observed around the cemented Oxford UKA [7]. Therefore, this led to the introduction of cementless version of Oxford UKA. The shape of the cementless prosthesis is identical to the cemented prosthesis, except the bottom surface having a porous titanium layer with calcium hydroxyapatite coating [8].

The cementless Oxford UKA is associated with a significant lower incidence of radiolucent lines than in cemented Oxford UKA, suggestive of an improved fixation [9]. Although there are several reports on the complications of the tibial side, such as valgus subsidence of the tibial component and tibial fractures, to the best of our knowledge, there have been no reported cases of complication on the femoral side of cementless Oxford UKA [10, 11]. Herein, we report two cases of femoral migration causing bearing dislocations after cementless Oxford UKA.

## Case presentation

### Case 1

An 82-year-old woman with anteromedial osteoarthritis of the right knee was treated with cementless Oxford UKA. The patient [body weight, 42 kg; height, 149 cm; and body mass index (BMI), 18.9 kg/m^2^] had undergone cemented Oxford on the left knee 3 years ago because cementless Oxford was not introduced at that time. Her preoperative Knee Society Score [12] was 51 points and the Knee Society Functional Score was 55 points. The range of motion (ROM) of the right knee was 140° flexion and 0° extension.

**Fig. 1:**
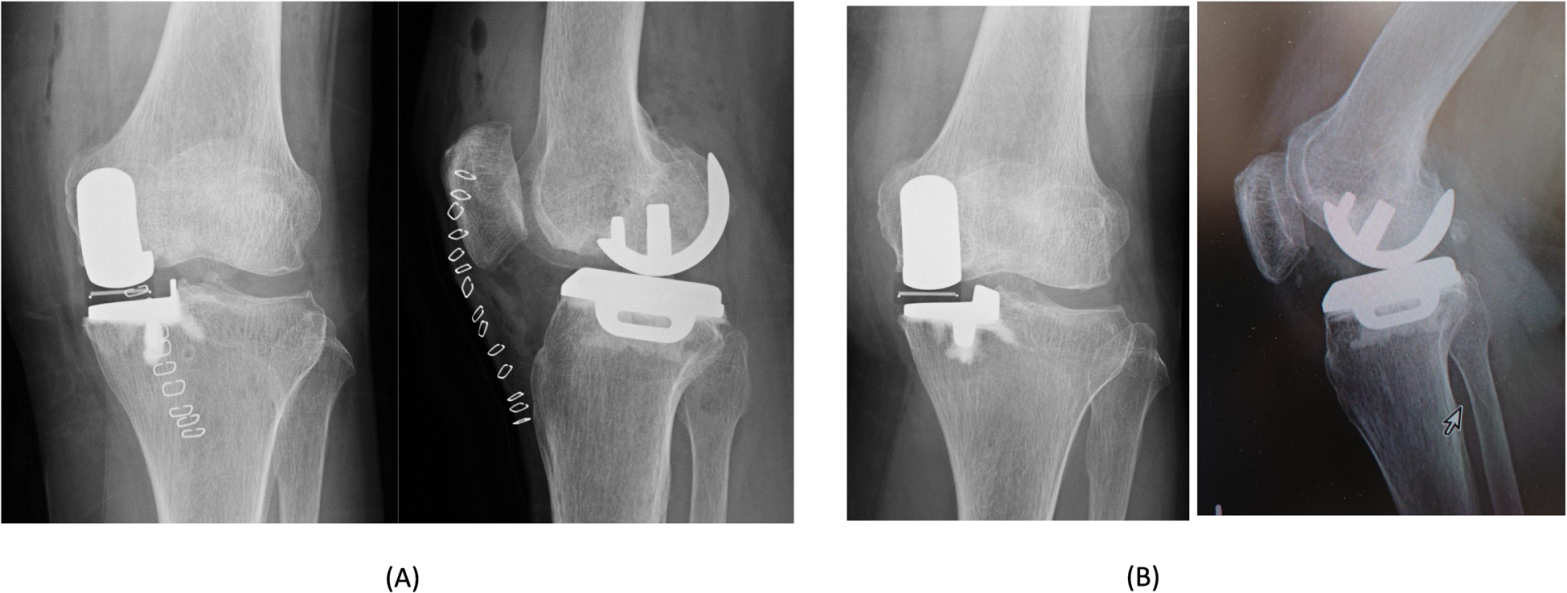
Case 1 radiographs. Postoperative plain radiographs of the left knee. **a** Postoperative radiographs at 1 year showing the migration of the femoral component (**b**)

During the surgery, a thigh tourniquet was applied and the leg was placed on a thigh support with the hip flexed about 30 degree. A minimally invasive approach was used. An incision was made from the medial pole of the patella to the medial side of the tibial tuberosity and was deepened through the joint capsule. At its upper end, capsular incision was extended proximally 1 cm into the vastus medialis. When performing osteotomy, tibial alignment was aimed at 90° to the mechanical axis in the frontal plane and 7° of the posterior slope in the sagittal plane, and femoral alignment was aimed at 90° to the mechanical axis in the frontal plane and 10°of flexion in the sagittal plane using Microplasty system [13]. In addition, we performed the gap balancing and implantation technique after the osteotomy procedures. The thickness of the mobile bearing was selected using the feeler gauge. The gauge thickness was thought to be appropriate when natural tension in the ligaments was achieved. Under these circumstances the feeler gauge would slide in and out easily but will not tilt. The cementless UKA was planned by an experienced surgeon (HI). However, during the surgery, we used cemented tibial component because we thought that the tibial bone quality was not good. A small-sized cementless femoral component and an A-size tibial cemented component with a 4-mm thick meniscal bearing were implanted. Intraoperatively, we confirmed the same gap balance between knee flexion and extension, and the anterior cruciate ligament was well tensioned and covered with synovial membrane. There were no abnormal movements of the meniscal bearing and no signs of bearing dislocation. The postoperative radiographs showed appropriate implantation (Fig. 1a).

Regarding postoperative rehabilitation, range of motion exercise and walking exercises first with a crutch and then a walker, were started on the first postoperative day. At 3 weeks postoperatively, the patient was discharged from our hospital and completed the rehabilitation protocol with physiotherapists.

At one-year postoperative follow-up, she felt no pain, and the Knee Society and Knee Society Functional Scores were 95 and 100 points, respectively. The ROM of the right knee was 140° flexion and 0° extension. She did not experience knee instability during her daily routine. Although radiography of the frontal plane showed migration of the femoral component, the radiographs showed no apparent loosening the femoral component, (Fig. 1b).

**Fig. 2:**
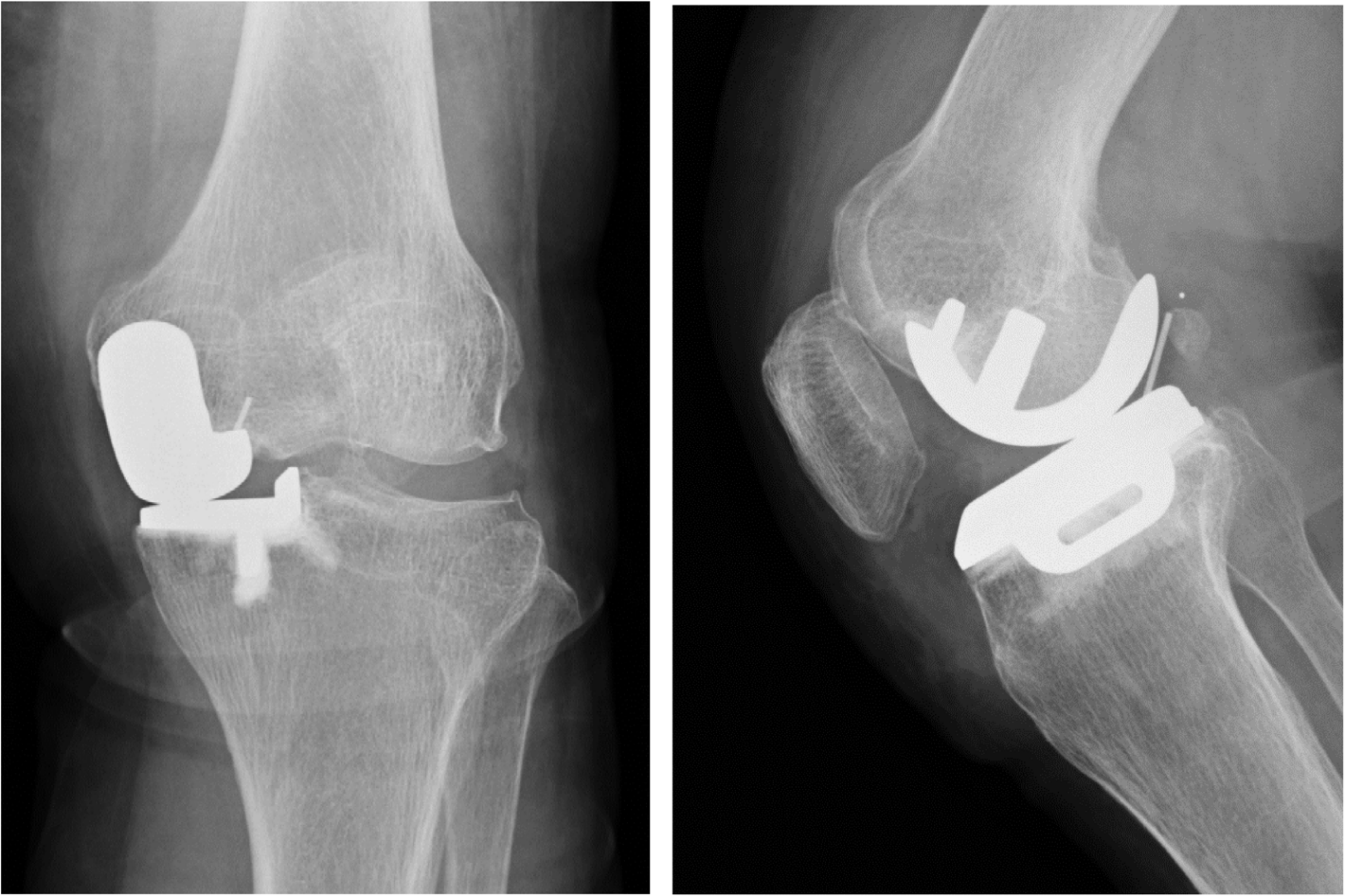
Radiographs of case 1 showing the bearing posterior dislocation

**Fig. 3:**
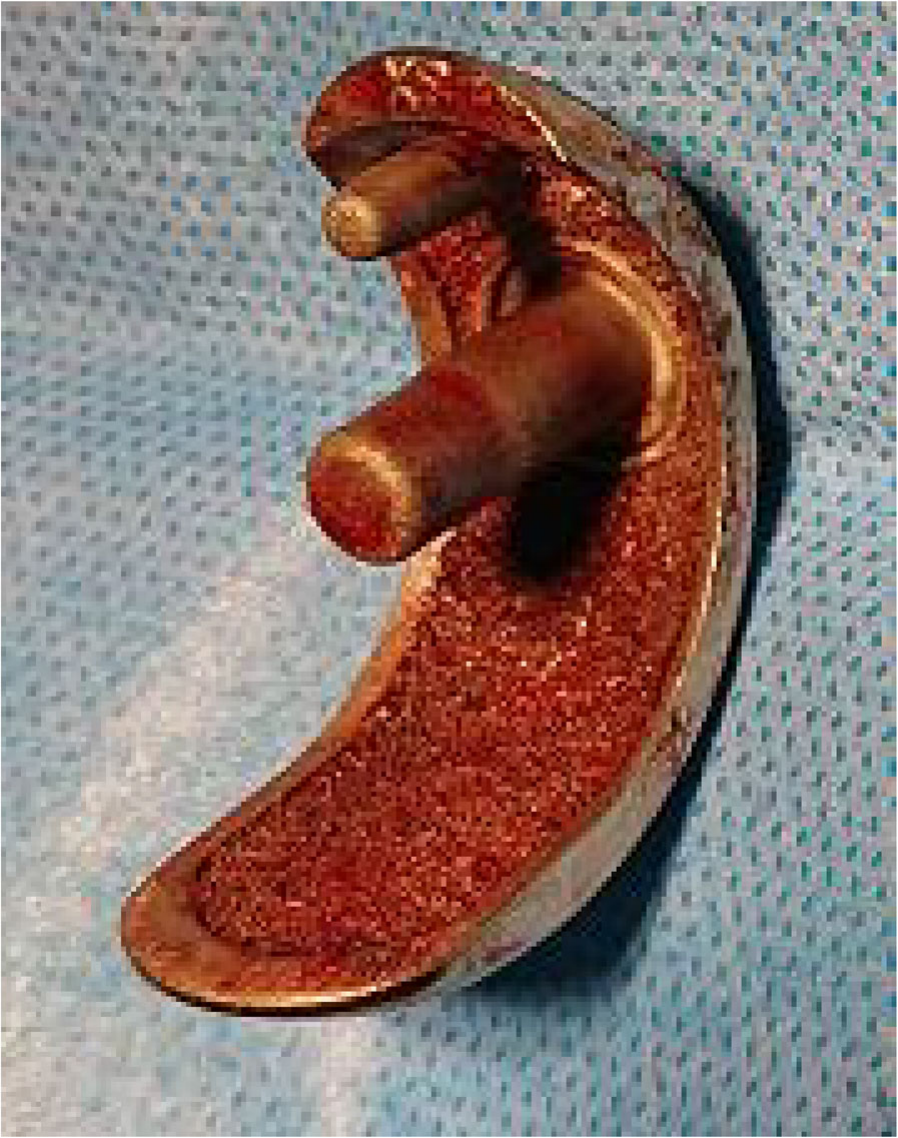
Image of the retrieved femoral component. No bone is attached to the undersurface of the retrieved femoral component

**Fig. 4:**
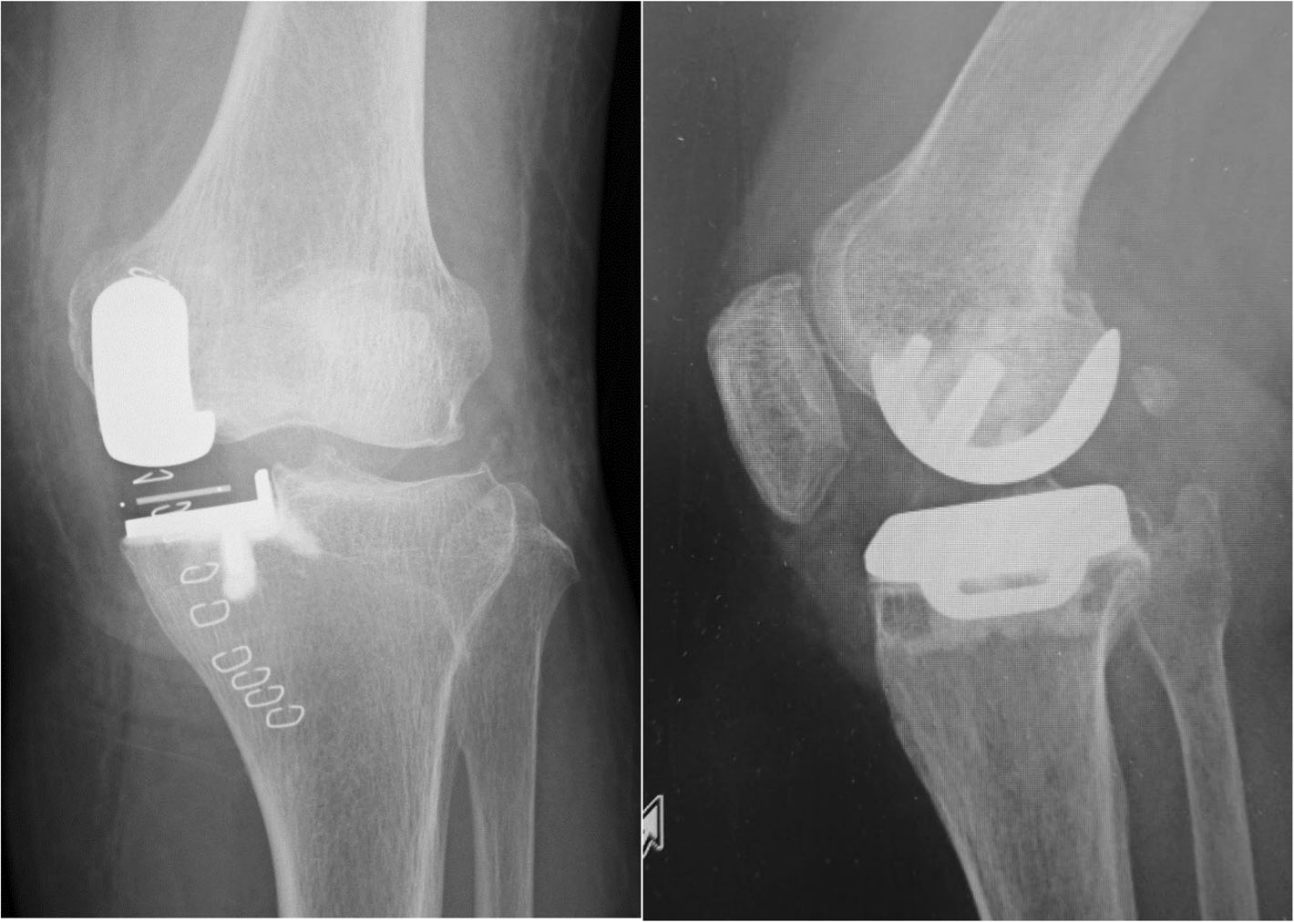
Radiographs of case 1 after the revision surgery

At 13 months postoperatively, she had sudden and severe knee pain while asleep. She could not walk, so she had to be brought to our facility in an ambulance. The meniscal bearing was posteriorly dislocated, as shown on the radiographic images (Fig. 2). We could not succeed in closed reduction; therefore, we performed surgical intervention. During the revision surgery, we observed the subsidence of the femoral component. The femoral component could easily be retrieved because of the loosening of the femoral component. In addition, there was no bone attached to the undersurface of the retrieved femoral component (Fig. 3). We implanted the same small-sized cemented Oxford femoral component, and the 8-mm thick bearing was inserted (Fig. 4). One year after the revision of UKA, her knee was pain-free and had a ROM of 0° extension to 140° flexion. She had no femoral migration and bearing re-dislocation.

### Case 2

**Fig. 5:**
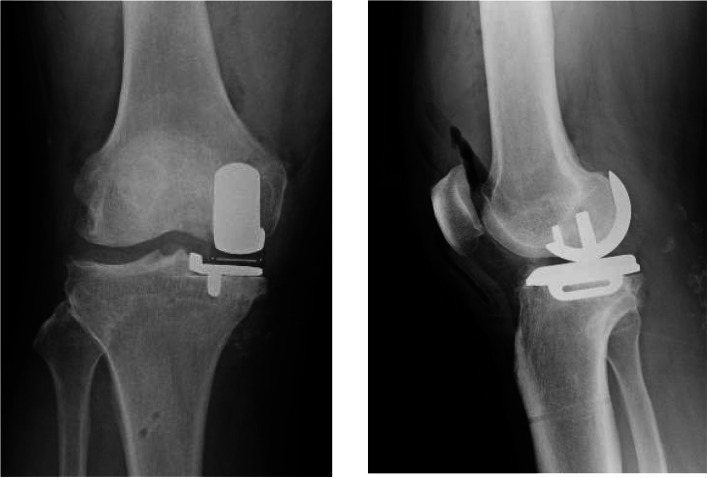
Case 2 radiographs. Postoperative plain radiographs of the left knee

**Fig. 6:**
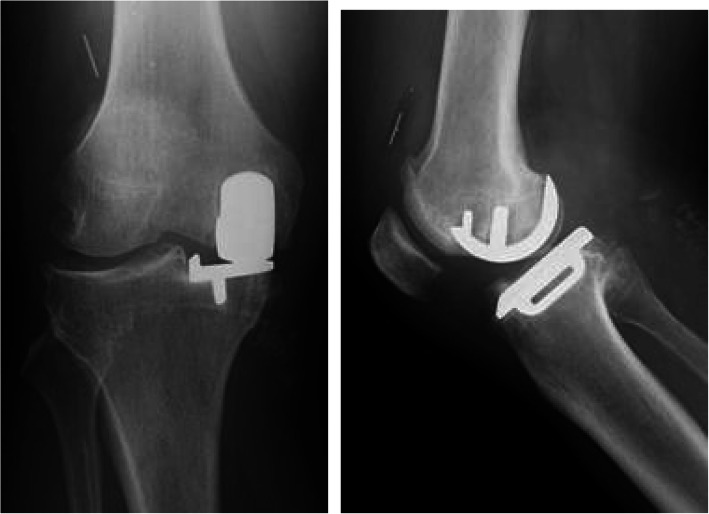
Postoperative radiographs of case 2 at 6 months showing bearing dislocation

**Fig. 7:**
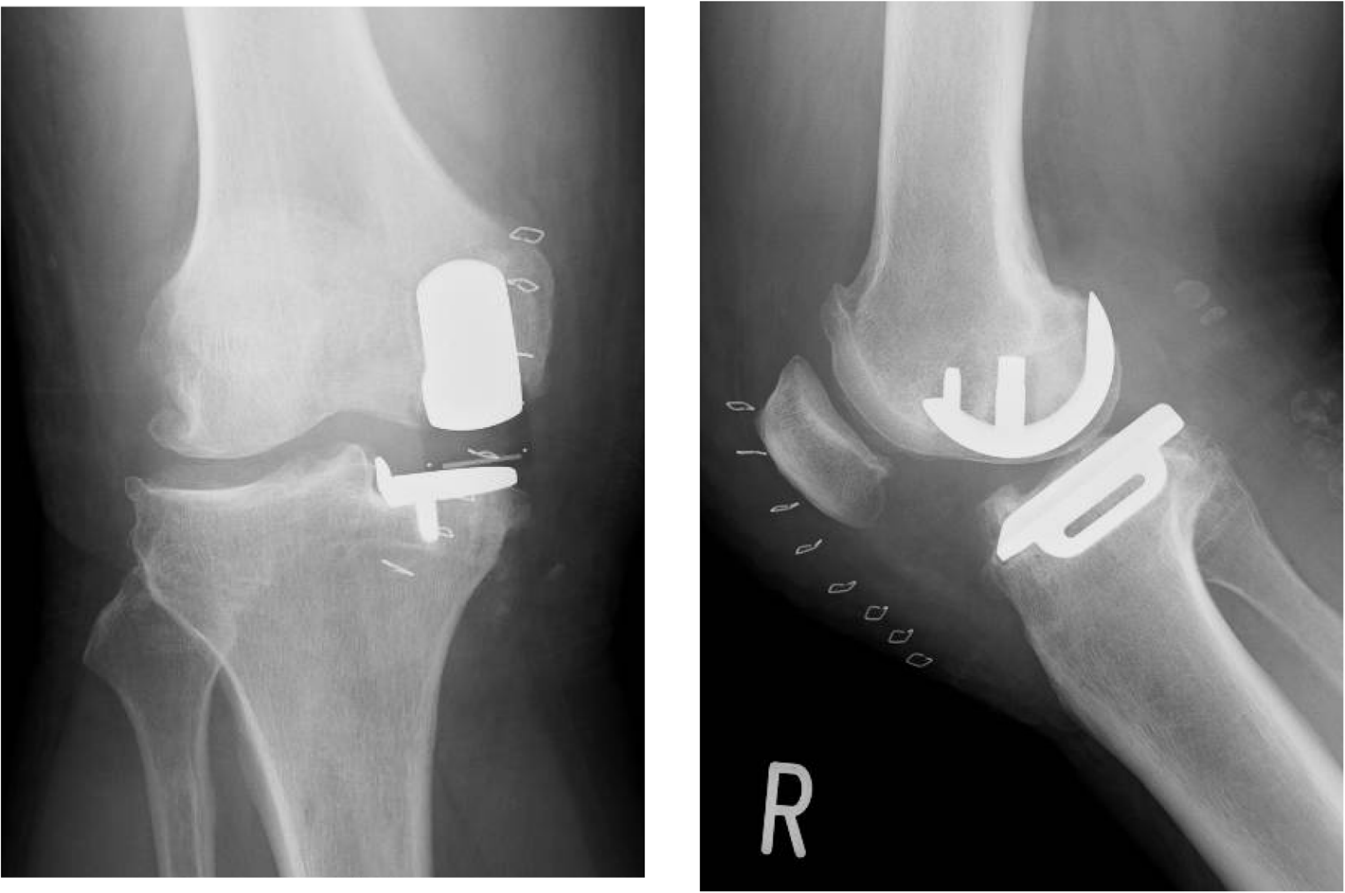
Radiographs of case 2 after the additional surgery showing femoral migration and radiolucent zone around the femoral pegs

**Fig. 8:**
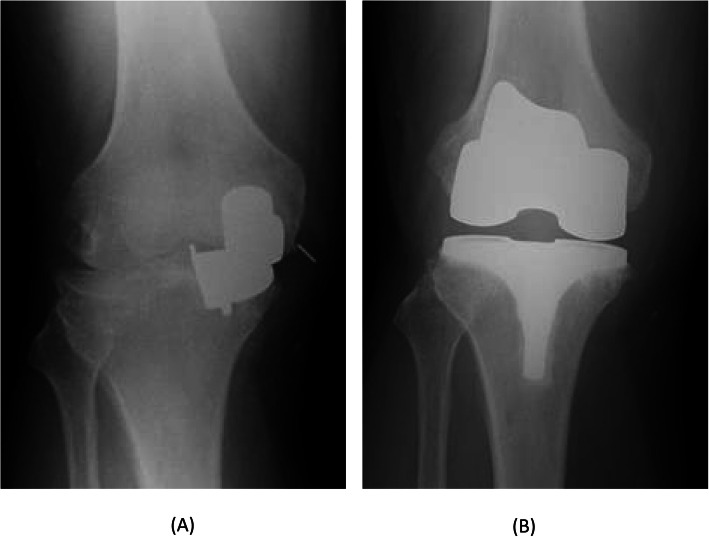
Radiographs 5 months after the additional surgery shows bearing re-dislocation (**a**). Radiographs after the revision to total knee arthroplasty (**b**)

The second case was a 52-year-old man (body weight, 101 kg; height, 179 cm; and BMI, 31.5 kg/m^2^) with anteromedial osteoarthritis of the right knee. The preoperative Knee Society and Knee Society Functional Scores were 57 and 60 points, respectively. The ROM of the left knee was 125° flexion and 5° extension. The surgical technique used for Case 2 was the same as that used for Case 1. The patient had a good bone quality. Therefore, a medium-sized cementless femoral component and D-sized cementless tibial component with a 5-mm thick meniscal bearing were implanted. Intraoperatively, we confirmed the same gap balance between knee flexion and extension, and the anterior cruciate ligament was well-tensioned and covered with synovial membrane. There was no abnormal movement of the meniscal bearing or signs of bearing dislocation. The postoperative radiographs showed appropriate implantation (Fig. 5). Immediately after the operation, the pain disappeared, and he resumed playing Judo 6 weeks postoperatively. Seven months postoperatively, he had sudden and severe knee pain when he tried to ride a bicycle. The meniscal bearing was anteriorly dislocated as shown on the radiographic images (Fig. 6). During the additional surgery, although the tibial component slightly subsided anteriorly, there was no loosening of the tibial component. We did not check the fixation stability of the femoral component because we did not expect loosening of the femoral component. We changed the mobile bearing from 5- to 9-mm thickness and confirmed no signs of bearing dislocation intraoperatively. We observed femoral migration and radiolucent zone around the femoral pegs from the radiographic images taken postoperatively (Fig. 7). Five months after the additional surgery, he experienced another bearing anterior dislocation, so we performed revision surgery (Fig. 8a). During the surgery, the femoral component subsided approximately up to 4 mm and was loose enough to be easily retrieved using an elevator. We retrieved the components and revised to total knee arthroplasty (Journey II BCS. Smith and Nephew, Memphis, TN, USA; Fig. 8b).

Judging from the clinical findings, laboratory data, the synovial cell count, and culture results of arthrocentesis of both cases, the onset of septic loosening was thought to be negative.

Both patients provided their consent for publication of their data.

## Discussion and conclusion

To the best of our knowledge, this is the first report on the femoral migration of cementless Oxford causing bearing dislocations. We have performed 345 Oxford UKAs since 2011, and cementless femoral component has been used for 113 knees since 2016. Regarding the bearing dislocations, we experienced three cases (2.7%) using cementless femoral prosthesis. One was a dislocation into the condylar ridge, whereas the other two cases were anterior and posterior bearing dislocations as mentioned in the present report. Conversely, among the 232 cases using cemented femoral component, we experienced only one bearing dislocation (0.5%) into the condylar ridge and no anterior or posterior dislocations.

The causes of bearing dislocations into the condylar ridge are the inappropriate combination of the component sizes (small femur and AA-size tibia) and diagnosis (osteonecrosis), but no technical error [14]. Moreover, we experienced no anterior and posterior dislocation cases in the cemented Oxford series. Therefore, we confirmed that our surgical procedure of Oxford UKA was appropriate.

However, we experienced two cases of anterior and posterior bearing dislocations after using cementless prosthesis. During the revision surgery, we could not find any cause, including ligament damage, component malposition, and unretrieved bony spur or free body, other than the migration of the femoral components. We think that excessive migration of the femoral components enlarged the gaps between the femoral and tibial components and caused the dislocations of mobile bearing.

The cause of femoral migration was speculated to be poor bone quality in Case 1. We didn’t perform the evaluation of bone quality preoperatively. After the revision surgery, dual-energy X-ray absorptiometry examination revealed a femoral neck bone mineral density of 0.386 mg/cm^2^ and the young adult mean of 68%. Eckert et al. [15] have reported that bone quality is the key decision factor for selecting cemented or cementless UKA implant. Kerens et al. [8] reported that in their 60 consecutive cementless Oxford cases, they gave up using cementless femoral prosthesis in 8 cases because of inferior bone quality and implanted cemented prosthesis. Since our introduction of cementless Oxford in 2016, we have used cementless tibial component in about 30% cases because the press-fit area in the cementless fixation is relatively small, and we are anxious about early loosening, particularly in patients with inferior bone quality. Conversely, we used cementless femoral prosthesis for almost all cases because the rigid fixation contributed by the two pegs and wide contact area between the bone and undersurface of the femoral component, which has a porous titanium layer with calcium hydroxyapatite coating. However, the femoral migration in Case 1 revealed the importance of the bone density of the femoral condyle.

Therefore, we should avoid using cementless prostheses for the femoral component, as well as for the tibial component, in those patients whose bone quality is poor.

In Case 2, it is difficult to surmise the causes because the patient was young and had a good bone quality. However, we speculate that one of the main reasons was his rapid return to aggressive sports. In fact, he was a famous Judo instructor and occasionally played Judo with Olympic level athletes. Kendrick et al. [16] have reported increased femoral migration of cementless Oxford up to 6 months, and thereafter, no significant additional migration. Excessive stress can enhance the risk of increased migration within 6 months postoperatively. Although Panzram et al. [17] reported 17 and 24 of 27 patients returned to sports within 3 and 6 months, respectively, after cementless Oxford UKA, we think that patients should not return to high performance sports until 6 months postoperatively, when migration reaches a plateau. Furthermore, obesity might be another reason for the migration. Obesity is a contraindication of Oxford UKA [18]; however, it is possible that obesity, in addition to early return to sports, may worsen the migration and lead to loosening and bearing dislocation.

Regarding the migration and subsidence of the tibial side of the cementless Oxford, several reasons other than poor bone quality and early return to sports have been reported, including impingement of the mobile bearing on the tibial wall, damage to the lateral part of the horizontal tibial surface, fracture of the posterior cortex during keel cut and tibial component malpositioning [10, 19]. Therefore there might be some other reasons of the femoral migration of the current report.

There are several merits of employing cementless Oxford UKA, including shorter operative time, equivalent or superior clinical outcomes, and improved long-term fixation at the expense of short-term fixation [8, 9, 15]. Therefore, we currently use cementless Oxford UKA for the patients who are not diagnosed with osteoporosis or who do not wish an early return to high-level physical activity. We think we should use cemented Oxford for the patients who need rigid fixation just after the UKA procedure. Recently, we have not experienced the migration of cementless prosthesis in any case. However, further observations are necessary to decide the ideal inclusion criteria for using cementless Oxford UKA.

## Data Availability

The datasets used during the current study are available from the corresponding author on reasonable request.

## Abbreviation

UKA: unicompartmental knee arthroplasty
